# Supplementary material for: The stress sensor GCN2 differentially controls ribosome biogenesis in colon cancer according to the nutritional context
Source: Mol Oncol. 2023 Aug 18;18(9):2111–35. doi: 10.1002/1878-0261.13491 (PMC11467793; doi:10.1002/1878-0261.13491)
Supplement: Supplementary file 1 — Fig. S1. Enrichment assessment of gene sets and signatures of interest in various types of cancers. Fig. S2. GCN2 accumulation and prognostic value in human colon adenocarcinoma. Fig. S3. Validation of the ISR impairment by the GCN2i. Fig. S4. Illustration of patient‐derived tumoroids grown in Matrigel® or as a colosphere in ultra‐low attachment plate. Fig. S5. Lack of GCN2 activity triggers a TP53‐dependent cell death upon low glutamine. Fig. S6. Lack of GCN2 activity impairs cell proliferation and spheroid formation. Fig. S7. Lack of GCN2 activity induces an autophagic flux and mTORC1 inhibition in HT29 cells grown in nutrient‐rich conditions. Fig. S8. Lack of GCN2 activity sensitizes COAD cells to RNA pol I inhibitors. Table S1. List of human primers used for RT‐qPCR experiments. Table S2. List of human probes used for Northern blotting. Table S3. List of gene signatures used in the present study. [file MOL2-18-2111-s001.pdf]

Supplementary information for:

The stress sensor GCN2 differentially controls ribosome biogenesis in colon cancer according to the nutritional context

Marie Piecyk[1,] Mouna Triki[1], Pierre-Alexandre Laval[1], Cedric Duret[1], Joelle Fauvre[1], Laura Cussonneau[2], Christelle Machon[1,3], Jerôme Guitton[1,3], Nicolas Rama[1], Benjamin Gibert[1], Gabriel Ichim[1], Frederic Catez[1], Fleur Bourdelais[1], Sebastien Durand[1], Jean-Jacques Diaz[1], Isabelle Coste[1], Toufic Renno[1], Serge N Manié[1], Nicolas Aznar[1], Stephane Ansieau[1], Carole Ferraro-Peyret[1,4] and Cedric Chaveroux[1]*

Affiliations:

[1] Centre de Recherche en Cancérologie de Lyon, INSERM U1052, CNRS 5286, Centre Léon Bérard, Université de Lyon, Université Claude Bernard Lyon 1, Lyon, France.

[2] Université Clermont Auvergne, INRAE, Unité de Nutrition Humaine, UMR1019, Clermont-Ferrand, France

[3] Biochemistry and Pharmaco-toxicology laboratory, Lyon Sud Hospital, University Hospital of Lyon, Pierre-Bénite, France.

[4] Hospices Civils de Lyon, Plateforme AURAGEN, Lyon, France.

Corresponding author:

*To whom correspondence should be addressed.

Email: cedric.chaveroux@lyon.unicancer.fr. Address: Cancer Research Center of Lyon, Inserm UMR 1052 CNRS 5286, Cheney D, floor 6, Centre Léon Bérard, 28 rue Laënnec, 69008 LYON, France. Tel: +33 (0)4 69 16 66 22

Supplementary information includes:

- Figure supplement S1-S8
- Supplementary movie 1
- Supplementary tables 1-3

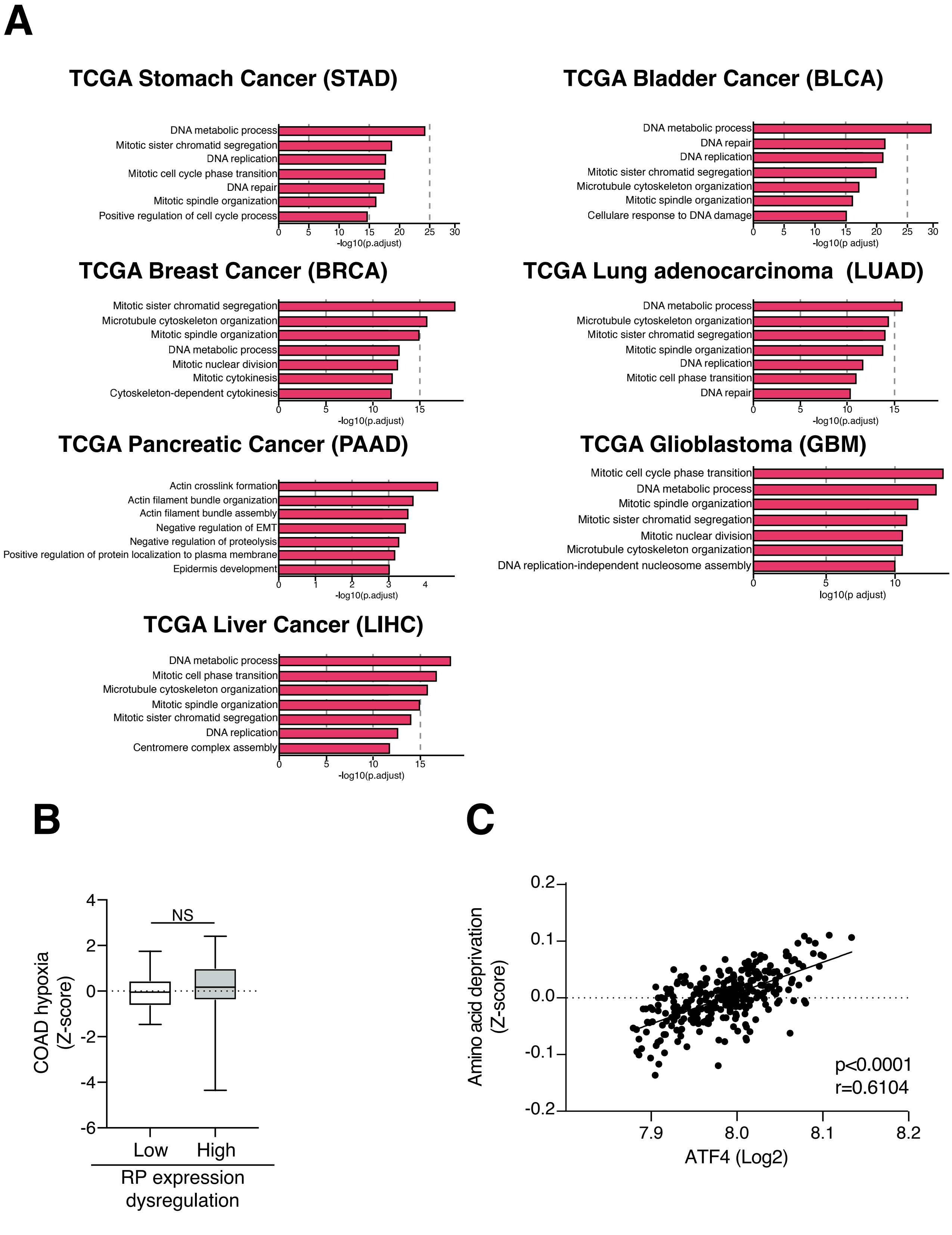

Figure supplement 1

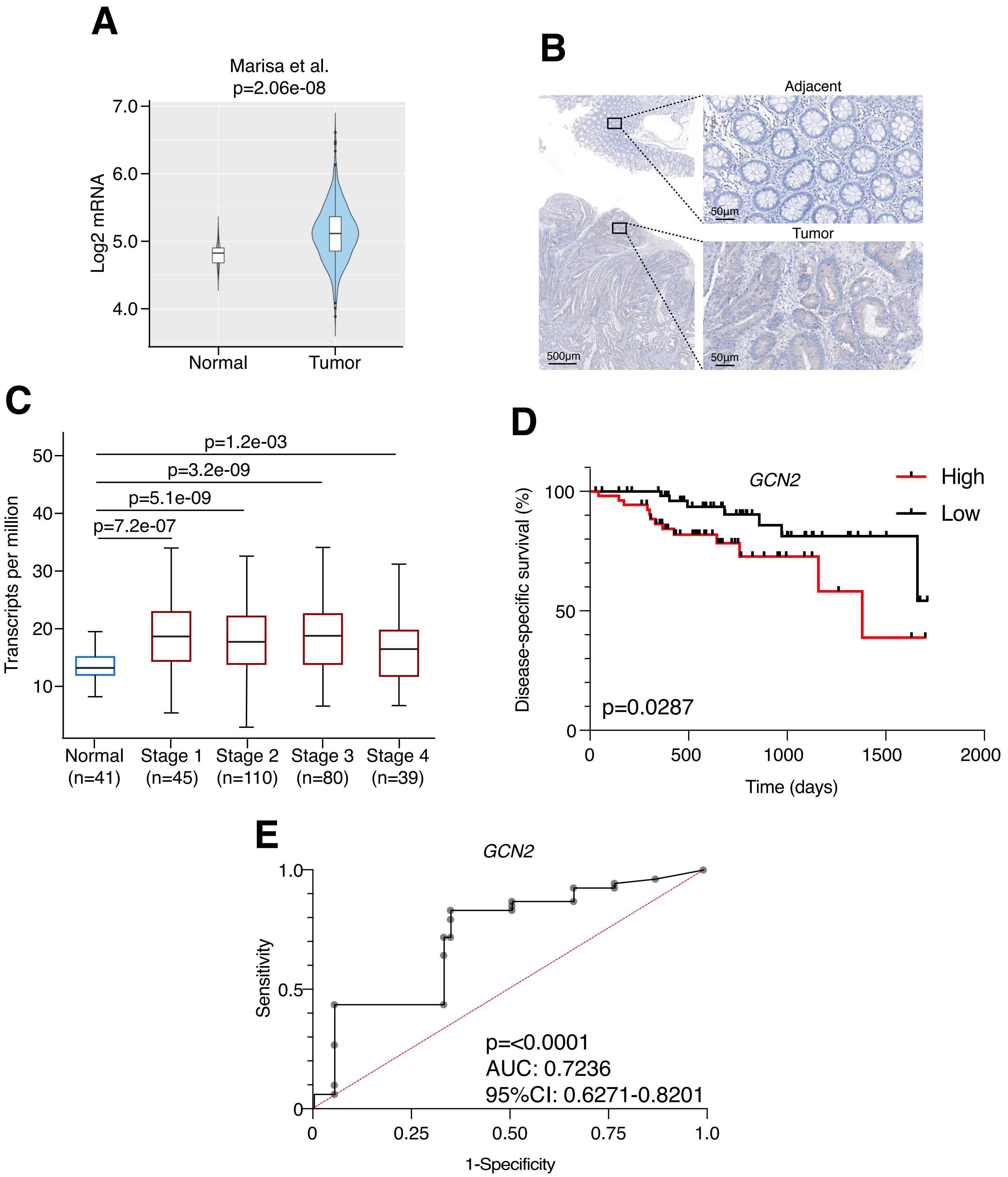

Figure supplement 2

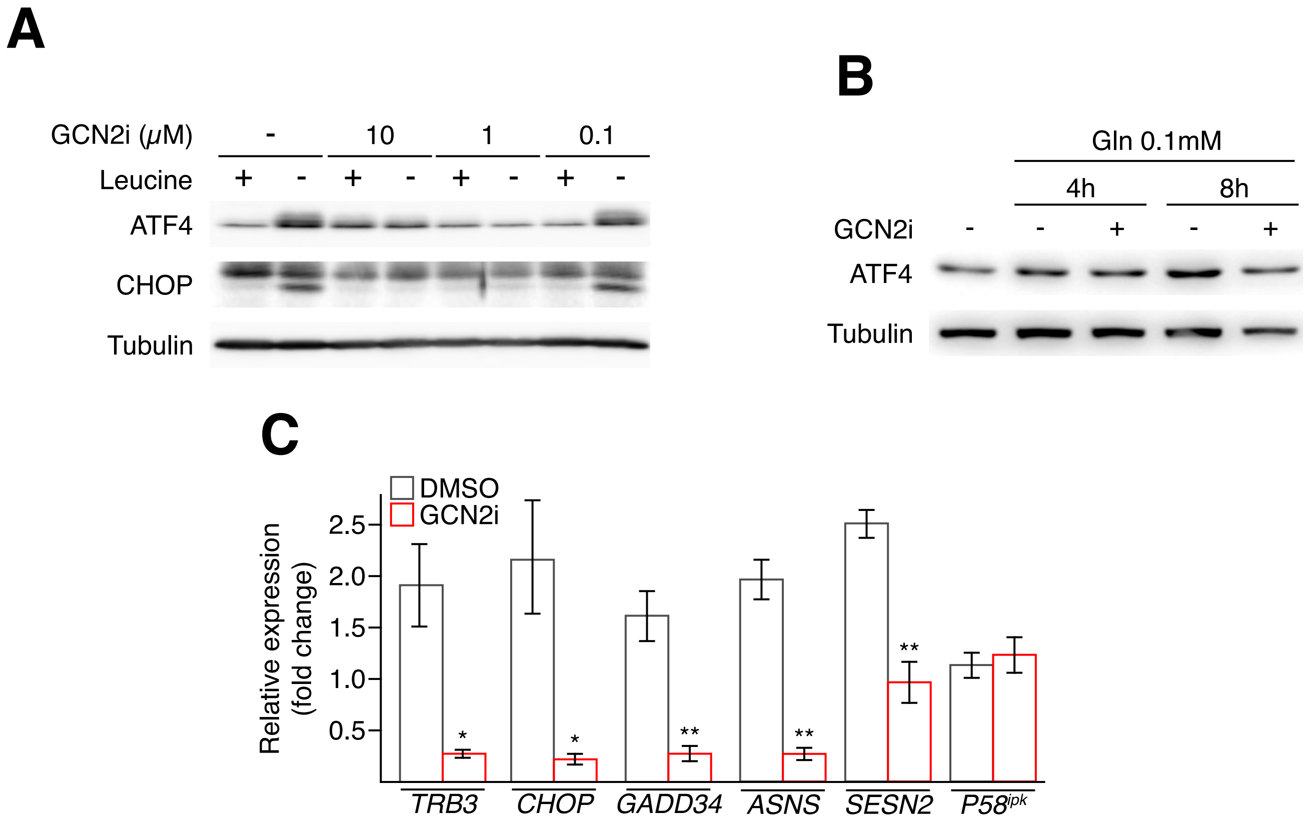

Figure supplement 3

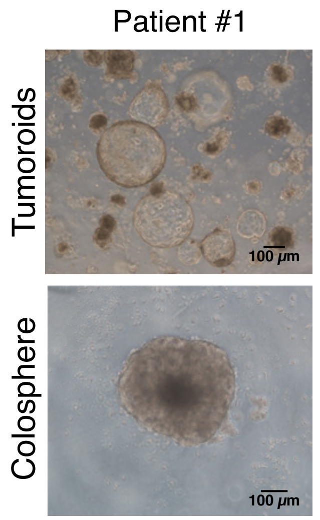

Figure supplement 4

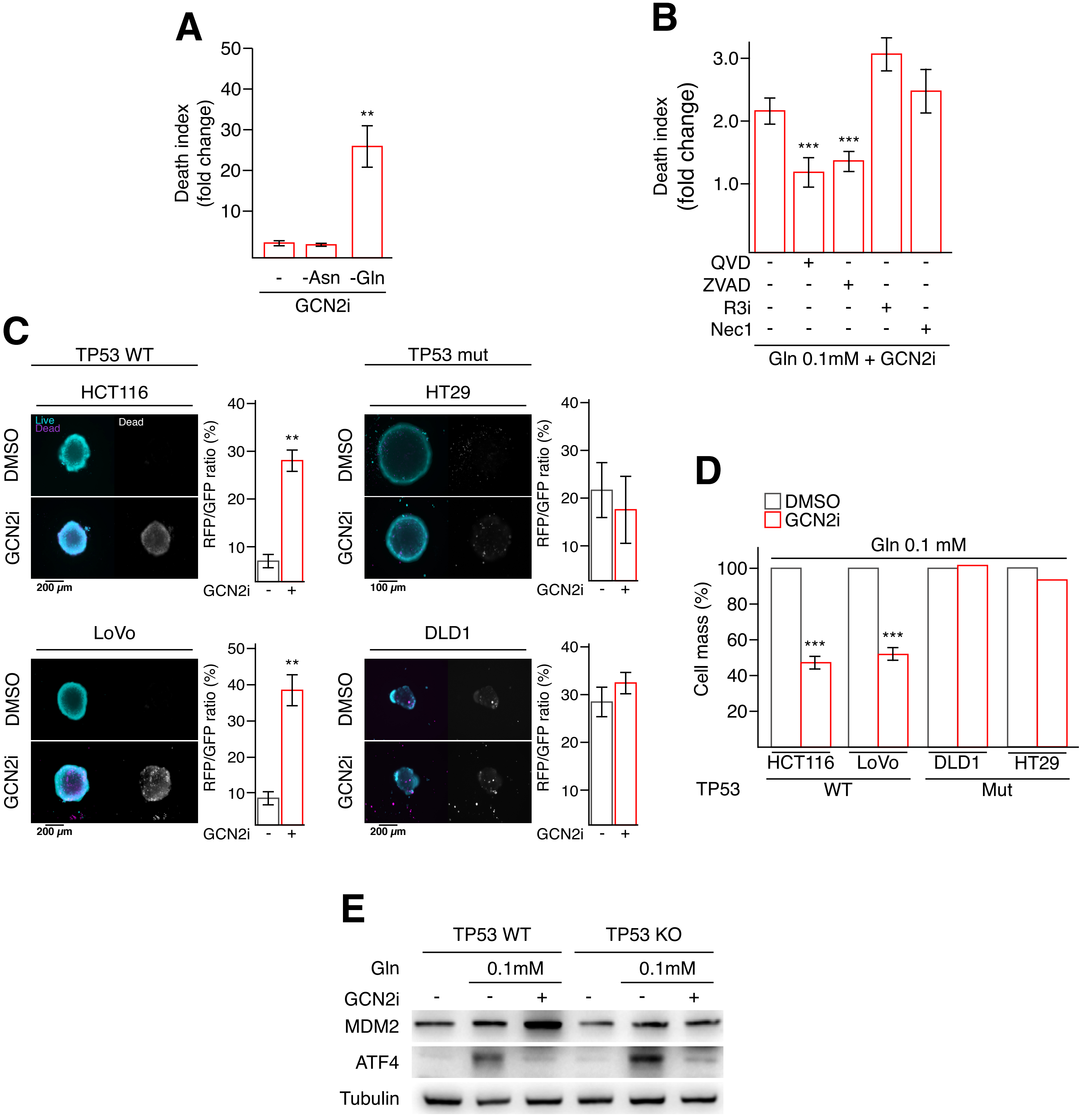

Figure supplement 5

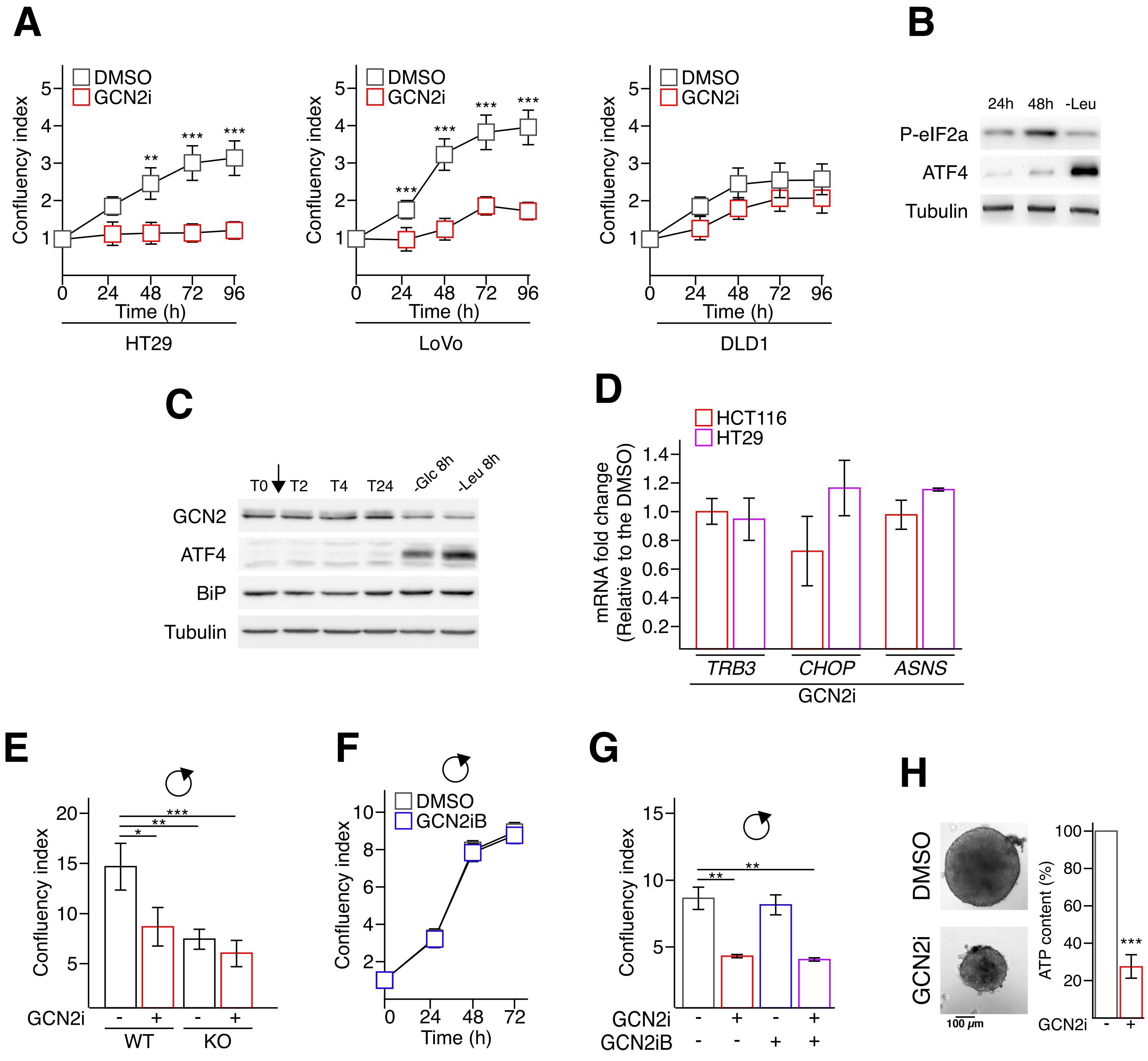

Figure supplement 6

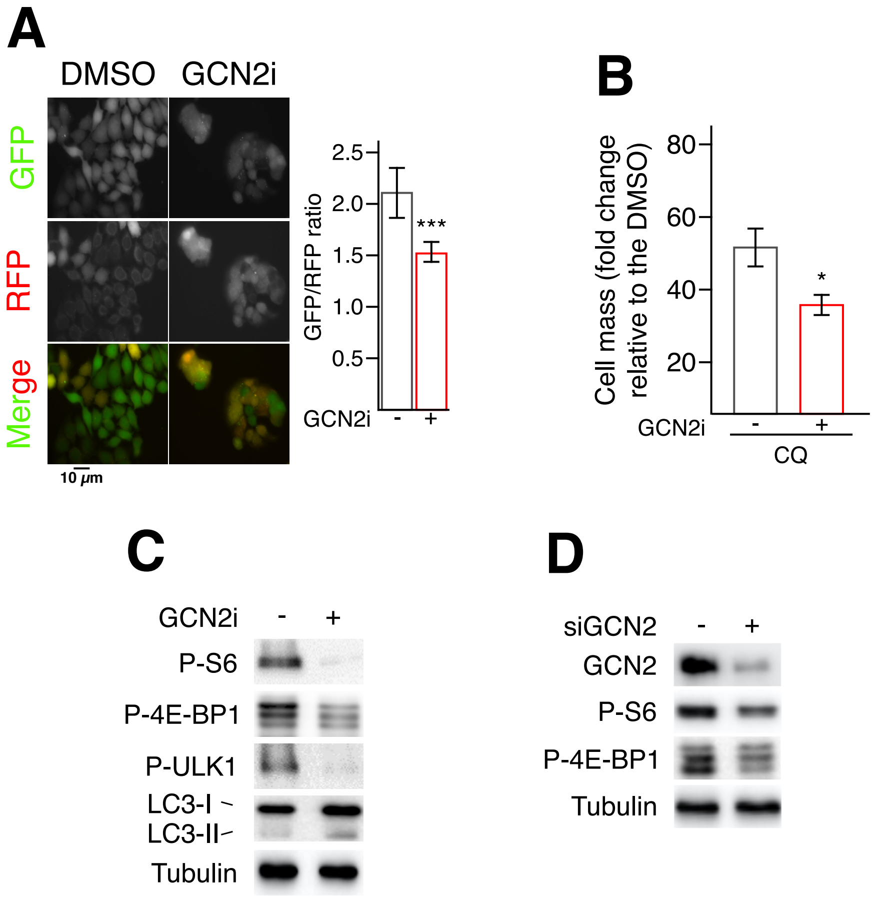

Figure supplement 7.

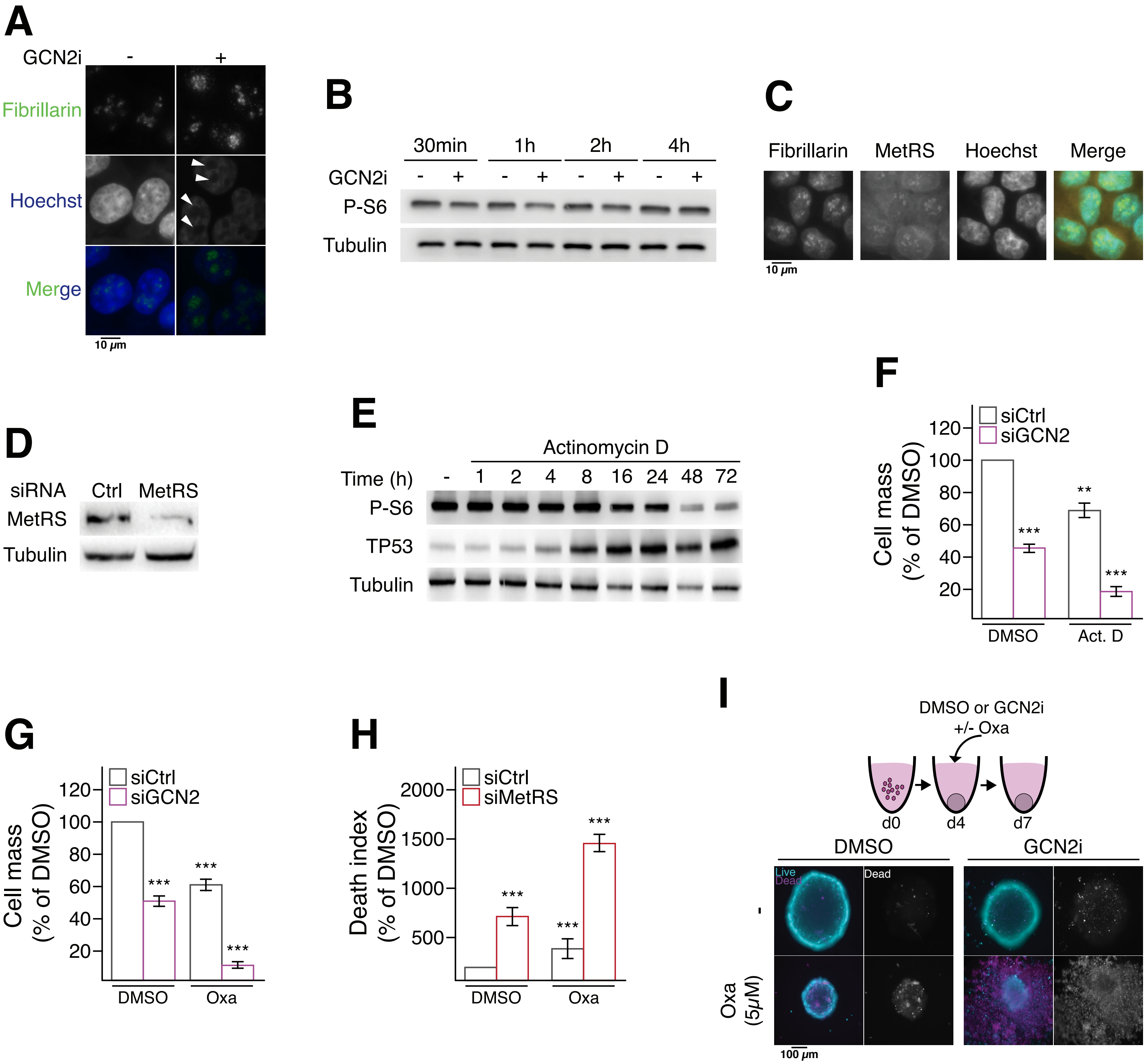

Figure supplement 8

ALDH1A3	Fw: CTGCTACAACGCCCTCTATGCA<br>Rev: GTCGCCAAGTTTGATGGTGACAG
c-MYC	Fw: CCTGGTGCTCCATGAGGAGAC<br>Rev: CAGACTCTGACCTTTTGCCAGG
TRB3	Fw: TGGTACCCAGCTCCTCTACG<br>Rev: GACAAAGCGACACAGCTTGA
ASNS	Fw: CTGTGAAGAACAACCTCAGGATC<br>Rev: AACAGAGTGGCAGCAACCAAGC
P58IPK	Fw: GCTGAATGTGGAGTAAATGCAG<br>Rev: GTGCAGCTTTTGATTTGCCC
SESN2	Fw: AGATGGAGAGCCGCTTTGAGCT<br>Rev: CCGATGAAGTCCTCATATCCG
CHOP	Fw: GGTATGAGGACCTGCAAGAGGT<br>Rev: CTTGTGACCTCTGCTGGTTCTG
GADD34	Fw: CTGTGATCGCTTCTGGCA<br>Rev: GGAAGAAAGGGTGGGCATC
47S	Fw: GTGCGTGTCAGGCGTTC<br>Rev: GGGAGAGGAGCAGACGAG
RPS11	Fw: TGACCTTGATTTATTTTGCATACC<br>Rev: CGAGCAAGACGTTCAGTCCT

Supplementary table 1

Actin	CGGTAGGACGCAGACCTGGACCGACCGGCCCTGG
5'ETS	CGACTGTGCGACAGGAGACCGCTGGACAGCAGCCTCTCCAACCCGGAGGC
ITS2	ACGCGCCGACCCCCAAGGGAGCGTC

Supplementary table 2

**COAD ribosome dysfunction**	**Amino acid deprivation**	**COAD Hypoxia**	**ATF4**
Negatively-regulated genes	ASNS	BCCIP	DDIT4
RPL34	ASS1	BNIP3L	NARS1
RPL9	ATF3	GADD45B	PSAT1
RPL21	ATF5	INSIG2	SREBF1
RPS25	CARS1	MPHOSPH6	IARS1
RPL3	CBS	TP53	IARS2
RPL11	CCNG2		MTHFD2
RPL26	CDKN1A		EIF2AK4
RPL3L	CEBPB		ATF3
RPS4Y2	CHAC1		GFPT1
RPL15	CLEC7A		WARS
RPL22	CTH		WARS2
RPS4Y1	CXCL8		NFE2L2
RPL10L	DDIT3		YARS
RPS27L	DDIT4		YARS2
RPL36AL	FYN		
FAU	GADD45A		
RPS29	MARS		
RPS26	PPP1R15A		
RPL15	PSAT1		
RPS12	RETN		
RPL10A	SARS		
RPS27A	SESN2		
RPS27	SLC38A2		
RPS13	SLC7A11		
RPS23	STC2		
RPS3A	TRIB3		
RPL17	VEGFA		
RPL39L	WARS		
Positively-regulated genes			
RPL36A			
RPS2			
RPL22L1			
RPL8			
RPL28			
RPLP0			
RPS19			

Supplementary table 3

Supplementary information legends

Figure supplement 1. Enrichment assessment of gene sets and signatures of interest in various types of cancers.
A. Gene Ontology enrichment analysis on TCGA database of different cancers.
B. Comparison of the COAD hypoxia gene signature in COAD cohorts with low and high levels of dysregulated riboproteins expression.
C. Correlation between amino acid deprivation and ATF4 gene signatures.

Figure supplement 2. GCN2 accumulation and prognostic value in human colon adenocarcinoma.
A. GCN2 mRNA levels in normal tissue compared to tumoral tissue. (Marisa et al. cohort)
B. Example of GCN2 amount accumulation in the tumor cells compared to the normal adjacent tissue.
C. Analysis of GCN2 expression accross normal tissue and the 4 stages of colon adenocarcinoma (TCGA database).
D. Disease specific survival of COAD patients with high (red) or low (black) expression of GCN2 5 years after the diagnosis (TCGA data).
E. ROC curve analysis of overall survival prediction based on the high expression of GCN2.

Figure supplement 3. Validation of the ISR impairment by the GCN2i.
A. Western blot analysis of ISR markers induction (ATF4 and CHOP) after 8h of leucine starvation (0.45mM versus 0mM), a classical inducer of the GCN2 pathway, with or without GCN2i at the indicated concentrations. Tubulin served as a loading control.
B. Western blot analysis of ATF4 after 8h of glutamine deprivation (Gln 2mM versus 0.1mM) with or without GCN2i at the indicated time points. Tubulin served as a loading control.
C. RT-qPCR analysis of canonical ATF4 target genes (TRB3, CHOP, GADD34, ASNS, SESN2) upon low glutamine in combination with DMSO or GCN2i. P58IPK is given as a non-ATF4 target gene.

Figure supplement 4. Illustration of patient-derived tumoroids grown in Matrigel® or as a colosphere in ultra-low attachment plate.
Microscopy images of the morphology of patient's derived COAD cells grown as matrix-embedded cultures (tumoroids) or in ULA attachment plates (colospheres). Of note, primary cells grown as colosphere lead to a visible compaction in the core of the 3D structure.

Figure supplement 5. Lack of GCN2 activity triggers a TP53-dependent cell death upon low glutamine.
A. HCT116 cell-death analysis following 4 days of GCN2i treatment in complete (-, 2mM Gln, 0.34mM Asn) or starved for glutamine (-Gln, 0.1mM) or asparagine (-Asn, 0.02mM) media.
B. Impact of inhibitors of necroptosis (Nec1, R3i) or apoptosis (z-VAD, QVD) on GCN2i-induced cell death following a 48h deprivation of glutamine (0.1 mM)

C. Live-and-dead assays in HCT116, LoVo, HT29 and DLD1 spheroids treated for 3 days with vehicle (DMSO) or GCN2i. Enlargement of the spheroids core is provided to visualize cell death.
D. Cell mass measurement of HCT116, LoVo, DLD-1 and HT-29 cells cultured in 2D conditions and treated with DMSO or GCN2i for 48 h. Media were refreshed daily (black rounded arrow).
E. Western blot analysis of ATF4 and MDM2 amounts after 8h of glutamine starvation with or without GCN2i, in HCT116 wild-type (WT) or knockout (KO) for TP53. Tubulin served as a loading control.

Figure supplement 5. Lack of GCN2 activity triggers a TP53-dependent cell death upon low glutamine.
A. HCT116 cell-death analysis following 4 days of GCN2i treatment in complete (-, 2mM Gln, 0.34mM Asn) or starved for glutamine (Gln, 0.1mM) or asparagine (Asn, 0.02mM) media.
B. Impact of inhibitors of necroptosis (Nec1, R3i) or apoptosis (z-VAD, QVD) on GCN2i-induced cell death following a 48h deprivation of glutamine (0.1 mM)
C. Live-and-dead assays in HCT116, LoVo, HT29 and DLD1 spheroids treated for 3 days with vehicle (DMSO) or GCN2i. Enlargment of the spheroids core is provided to visualize cell death.
D. Cell mass measurement of HCT116, LoVo, DLD-1 and HT-29 cells cultured in 2D conditions and treated with DMSO or GCN2i for 48 h. Media were refreshed daily (black rounded arrow).
E. Western blot analysis of ATF4 and MDM2 amounts after 8h of glutamine starvation with or without GCN2i, in HCT116 wild-type (WT) or knockout (KO) for TP53. Tubulin served as a loading control.

Figure supplement 6. Lack of GCN2 activity impairs cell proliferation and spheroid formation.
A. Cell proliferation assessed by the confluency index of HT29, LoVo and DLD1 cells treated or not with GCN2i for 96 hours.
B. Western blot analysis of P-eIF2a and ATF4 amounts after 24 and 48h of culture of HCT116 cells. HCT116 deprived for glucose (-Glc) or leucine (-Leu) for 8h were used as positive control. Tubulin served as a loading control.
C. Time course analysis of GCN2, ATF4 and BiP amounts in HCT116 cells. HCT116 deprived for leucine (Leu) or glucose (Glc) for 8h were used as positive controls. Tubulin served as a loading control.
D. RT-qPCR analysis of ATF4 target genes (TRB3, CHOP and ASNS) in HCT116 cells grown in complete medium and treated with DMSO or GCN2i for 48h.
E. Confluency measurement of immortalized MEFs wild type (WT) or knockout (KO) for GCN2 treated or not with GCN2i followng 48 hours of culture. Cell media were refreshed every 24 hours.
F. Cell proliferation measurement was assessed by the confluency index. HCT116 were treated or not with GCN2iB for 72 hours and media were refreshed every 24 hours.
G. Confluency measurement of HCT116 treated with GCN2i, GCN2iB or both for 48 hours of culture. Cell media were refreshed every 24 hours.

H. Spheroid formation assays of HT29 treated with DMSO or GCN2i. ATP content was measured after 7 days of treatment. Treatment was initiated concomitantly to plating.

Figure supplement 7. Lack of GCN2 activity induces an autophagic flux and mTORC1 inhibition in HT29 cells grown in nutrient-rich conditions.
A. Autophagic flux analysis using HT29 cells stably expressing the GFP-LC3-RFP-LC3ΔG construct and treated with GCN2i for 48 h. Cleavage of GFP-LC3 by autophagy released the RFP-LC3 as an internal control, thus reduction of the GFP/RFP ratio illustrates induction of the autophagic flux.
B. Cell mass was measured in GCN2i-treated HT29 cells after 48 h in combination with chloroquine. Data are expressed relative to the vehicle.
C. Western blot analysis of mTORC1 and autophagy markers (P-S6, P-4E-BP1, P-ULK1, LC3-I/II) following GCN2i treatment for 24h. Tubulin served as a loading control.
D. Western blot analysis of mTORC1 markers (P-S6, P-4E-BP1) following GCN2 silencing for 96h. Tubulin served as a loading control.

Figure supplement 8. Lack of GCN2 activity sensitizes COAD cells to RNA pol I inhibitors.
A. Fibrillarin localization assessed by immunofluorescence in HCT116 cells grown in complete DMEM with DMSO or GCN2i.
B. Western blot analysis of P-S6 in HCT116 cells treated with or without GCN2i at the indicated time points.
C. Immunocytofluorescence in HCT116 against fibrillarin and methionyl-tRNA synthetase (MetRS) associated to Hoechst coloration.
D. Western blot analysis of MetRS amount in HCT116 cells transfected for 72h with a siRNA control (Ctrl) or against MetRS.
E. Western blot analysis of P-S6 and TP53 in HCT116 cells treated with 1nM of actinomycin D at the indicated time points.
F. Cell mass measurement of HCT116 silenced or not for GCN2 and treated for 48 h with actinomycin D (Act. D).
G. Cell mass measurement of HCT116 silenced or not for GCN2 and treated for 48 h with oxaliplatin (Oxa).
H. Cell death measurement of HCT116 silenced or not for MetRS and treated for 48 h with oxaliplatin (Oxa).
I. Live-and-dead assays in HCT116 spheroids after 5 days of treatment with oxaliplatin (Oxa) and/or GCN2i. To facilitate the observation of cell death induction, staining of non-viable cells is also represented in shades of gray.

Supplemental movie 1. Live-imaging of cell death caused by the GCN2 inhibitor in combination with oxaliplatin.
Cell death visualization by incorporation of propidium iodide in HCT116 spheroids treated with the vehicle (DMSO) or GCN2 inhibitor (GCN2i) in combination or not with oxaliplatin (time course of treatments: 120 hours).

Supplementary table 1. List of human primers used for RT-qPCR experiments.

Supplementary table 2. List of human probes used for Northern blotting.

Supplementary table 3. List of gene signatures used in the present study.
